# TRIP13 impairs mitotic checkpoint surveillance and is associated with poor prognosis in multiple myeloma

**DOI:** 10.18632/oncotarget.14957

**Published:** 2017-02-01

**Authors:** Yi Tao, Guang Yang, Hongxing Yang, Dongliang Song, Liangning Hu, Bingqian Xie, Houcai Wang, Lu Gao, Minjie Gao, Hongwei Xu, Zhijian Xu, Xiaosong Wu, Yiwen Zhang, Weiliang Zhu, Fenghuang Zhan, Jumei Shi

**Affiliations:** ^1^ Department of Hematology, Shanghai Tenth People's Hospital, Tongji University School of Medicine, Shanghai 200072, China; ^2^ Shanghai Key Laboratory of Plant Functional Genomics and Resources, Shanghai Chenshan Botanical Garden, Shanghai 201602, China; ^3^ Shanghai Chenshan Plant Science Research Center, Chienes Academy of Sciences, Shanghai 201602, China; ^4^ Department of Internal Medicine, University of Iowa, Iowa City, IA 52242, USA; ^5^ CAS Key Laboratory of Receptor Research, Drug Discovery and Design Center, Shanghai Institute of Materia Medica, Chinese Academy of Sciences, Shanghai 201203, China

**Keywords:** TRIP13, multiple myeloma, prognosis, drug resistance, MAD2

## Abstract

AAA-ATPase TRIP13 is one of the chromosome instability gene recently established in multiple myeloma (MM), the second most common and incurable hematological malignancy. However, the specific function of TRIP13 in MM is largely unknown. Using sequential gene expression profiling, we demonstrated that high TRIP13 expression levels were positively correlated with progression, disease relapse, and poor prognosis in MM patients. Overexpressing human TRIP13 in myeloma cells prompted cell growth and drug resistance, and overexpressing murine TRIP13, which shares 93% sequence identity with human TRIP13, led to colony formation of NIH/3T3 fibroblasts *in vitro* and tumor formation *in vivo*. Meanwhile, the knockdown of TRIP13 inhibited myeloma cell growth, induced cell apoptosis, and reduced tumor burden in xenograft MM mice. Mechanistically, we observed that the overexpression of TRIP13 abrogated the spindle checkpoint and induced proteasome-mediated degradation of MAD2 primarily through the Akt pathway. Thus, our results demonstrate that TRIP13 may serve as a biomarker for MM disease development and prognosis, making it a potential target for future therapies.

## INTRODUCTION

Multiple myeloma (MM), a clonal neoplasm characterized by the expansion of malignant plasma cells in the bone marrow, is the second most common hematological malignancy worldwide. Despite the development of novel treatment agents, MM remains incurable for the majority of patients owing to drug resistance, which has prompted the need to identify novel therapeutic strategies.

As a heterogeneous disease, MM is typified by almost universal aneuploidy and recurrent chromosomal aberrations, leading to chromosomal instability (CIN) [1]. CIN has also been correlated with acquired or intrinsic drug resistance [2]. In addition, cellular mechanisms that normally enforce aneuploidy have been clarified in terms of defects in spindle checkpoints [3]. The kinetochore, a specialized protein/DNA-containing structure located at the centromere of each chromosome, keeps the paired sister chromatids together and tensioned by attaching to the mitotic spindles, which will pull the sister chromatids apart upon the onset of anaphase during mitosis [3]. Spindle checkpoint proteins associated with the kinetochore will arrest the mitotic program when sensing the lack of attachment and/or tension at the kinetochores, and this is part of a surveillance mechanism. These checkpoint proteins will swiftly become inactivated once all chromosomes are properly positioned. The role of spindle checkpoints is to maintain proper chromosome segregation and genetic stability by delaying metaphase-anaphase transition during cell division in the presence of defective kinetochore-microtubule attachment [4]. However, dysfunctions of checkpoint proteins allow cells to go through mitosis (transition from metaphase to anaphase) prematurely, resulting in chromosomal mis-segregation and aneuploidy in cancer-prone daughter cells [3].

Recently, we have characterized 10 CIN genes that are strongly correlated with MM drug resistance and disease relapse. Thyroid hormone receptor-interacting protein 13 (TRIP13), an AAA-ATPase, is one of these 10 CIN genes, which are significantly up-regulated in MM, especially in high-risk MM [5]. Also, according to gene expression profile (GEP) data, TRIP13 has been demonstrated to be one of the most important genes in the 70-gene high risk model of MM [6, 7]. However, GEP-based signature has limited power to predict therapeutic response in MM patients [8], highlighting the need to validate and specify the exact role of target genes *in vitro* and *in vivo*. Generally, TRIP13 is required for chromosome structure and recombination during mitosis [9], and is also a mitotic checkpoint-silencing protein [4], suggesting its regulatory effects on checkpoint proteins and its potential role in promoting cancers. Banerjee *et al*. have recently discovered that TRIP13-mediated drug resistance in squamous cell head and neck carcinoma occurs via enhanced double strand break (DSB) DNA repair through nonhomologous end joining signaling, which repairs the DSBs through the whole cell cycling [10]. However, the exact function of TRIP13 in the pathogenesis of MM has not been investigated yet.

In this study, we compare the TRIP13 expression in newly diagnosed and relapsed MM samples and correlate the TRIP13 expression with patient outcomes. We evaluate TRIP13 functions in MM cell growth, drug resistance and tumorigenesis *in vitro* and *in vivo*. Mechanistically, we determine that TRIP13 impairs checkpoint surveillance and identify a novel pathway that induces proteasome-mediated MAD2 degradation and drug resistance in MM. These data will help elucidate novel mechanisms responsible for tumorigenesis and treatment failure in MM and provide a potential new target in MM therapy.

## RESULTS

### TRIP13 is linked to myeloma progression, disease relapse, and poor prognosis in MM

**Figure 1:**
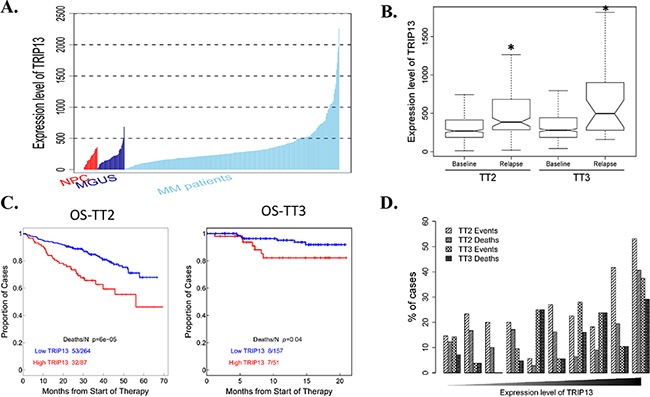
Gene expression profiling (GEP) analysis indicates TRIP13 is positively associated with myeloma development, disease relapse and poor prognosis in myeloma patients **A**. Expression level of TRIP13 in CD138-enriched plasma cells from 22 healthy subjects (NPC), 44 subjects with MGUS and 351 patients with newly diagnosed MM. Statistical significance of the differences in TRIP13 expression levels by t-test: MGUS vs. NPC, p = 0.65; MM patients vs. NPC, p< 0.01; MM patients vs. MGUS, p < 0.01. **B**. The expression level of TRIP13 was significantly up-regulated in relapsed patients from TT2 and TT3 cohort in comparison with patients at the baseline stage (*p < 0.05). **C**. Kaplan-Meier analyses of OS about patients from TT2 (p <0.001) and TT3 (p < 0.05) cohort revealed inferior outcomes among the patients with high TRIP13 expression compared with the remaining patients with low TRIP13 expression. **D**. The proportion of patients with MM events or deaths increased with the expression level of TRIP13. In each cohort, patients divided into 10 equal-sized groups based on the expression levels of TRIP13are shown on the x-axis from left to right. The relationships between the percentages of events/deaths and the expression level of TRIP13 showed general positive correlations (Pearson's correlation coefficient: TT2 events, r=0.72, p=0.018; TT2 deaths, r=0.51, p=0.13; TT3 events, r=0.78, p=0.0073; TT3 deaths, r=0.74, p=0.015).

*TRIP13* is one of the 10 CIN genes characterized in our previous study [5]. It has two isoforms: isoform 1 and isoform 2. The probes from Affymetrix U133 plus 2.0 microarray were identified specific to TRIP13 isoform 1, which has a longer C-terminus compared with isoform 2. Therefore, we focused on TRIP13 isoform 1 and its function in this study. We firstly compared TRIP13 expression levels in CD138-enriched plasma cells from 22 healthy subjects (normal plasma cells, NPC), 44 subjects with monoclonal gammopathy of undetermined significance (MGUS) and 351 patients with newly diagnosed MM. We did not see expression difference between NPC and MGUS (p=0.65), however, TRIP13 was significantly increased in newly diagnosed MM patients compared to NPC and MGUS samples (p<0.01) (Figure 1A). We also compared TRIP13 expression from 51 paired MM samples obtained at baseline (BL) and at relapse (RL) using GEP in total therapy 2 (TT2) and total therapy 3 (TT3). TRIP13 was significantly increased in relapsed MM samples compared to those collected at diagnosis (p < 0.01 in TT2, p < 0.05 in TT3) (Figure 1B). Next, we correlated the gene expression of TRIP13 with patient outcomes. We performed log-rank tests and presented with Kaplan-Meier survival curves between high (quartile 4) and low (quartiles 1 ∼ 3) samples from the TT2 and TT3 cohorts, which included 351 and 208 GEPs respectively. Results demonstrated that patients with high TRIP13 had inferior overall survival (OS) in both TT2 and TT3 trials (Figure 1C; p < 0.001 in TT2, p < 0.05 in TT3). From another perspective, when patients in each cohort were divided into 10 equal-sized groups on the basis of the ranked expression levels of TRIP13 (on the x-axis from left to right), the proportion of patients with either MM events or death was generally positively correlated to the expression levels of TRIP13 (Figure 1D).

### Overexpression of TRIP13 induces myeloma cell growth and drug resistance

**Figure 2:**
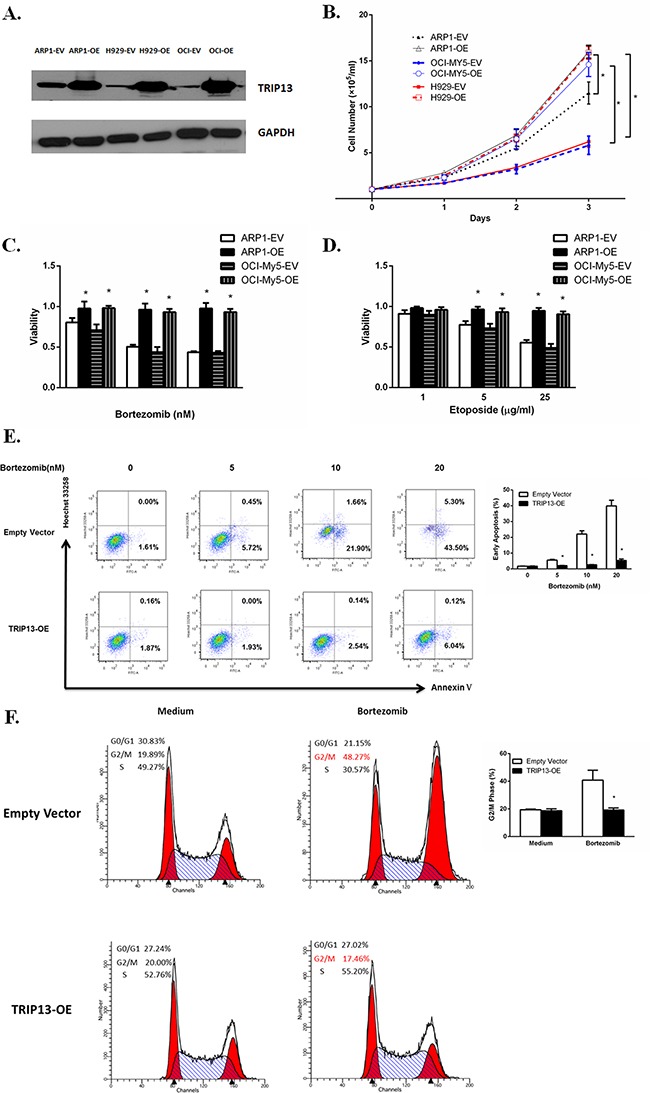
Increased TRIP13 induces cell growth and drug resistance **A**. TRIP13proteins were overexpressed inTRIP13 overexpressing (OE) MM cell lines ARP1, OCI-My5 and H929 compared to their counterparts transfected with empty vectors (EV). **B**. TRIP13-OE and EV transfected MM cell lines ARP1, OCI-My5 and H929 were counted by trypan blue exclusion for consecutive 3 days. High levels of TRIP13 significantly promoted myeloma cell growth. Results from 3 independent experiments are shown (EV vs. OE, *p < 0.05). **C**. and **D**. TRIP13-OE MM cell line ARP1 or OCI-My5 and their EV-transfected control cells were treated with indicated concentrations of proteasome inhibitor Bortezomib (C) or topoisomerase inhibitor Etoposide (D). Cell viability assay was performed 24 h later using PrestoBlue Cell Viability reagent. Results from 3 independent experiments are shown (*p<0.05). **E**. FITC-conjugated Annexin V/Hoechst 33258 staining was performed on TRIP13-OE MM cell line ARP1 or empty vector-transfected cells 18 h post different doses of Bortezomib treatment by flow cytometry (FCM). Cells that are in early apoptosis are Annexin V positive and Hoechst 33258 negative. Representative pictures of FCM were shown (left panel) with quantification of percentage of cells with early apoptosis (right panel). Results from 3 independent experiments are shown (*p < 0.05). **F**. TRIP13-OE MM cell line ARP1 or empty vector-transfected cells were treated with 20nMBortezomib for 24 h. Then cells were stained with propidium iodide (PI) and assayed for DNA content by FCM. TRIP13 overexpression lessened the Bortezomib-induced G2/M cell cycle arrest in myeloma cells compared to control cells. Representative pictures of FCM were shown (left panel) with quantification of percentage of cells at G2/M phase (right panel). Results from 3 independent experiments are shown (OE vs. EV, *p < 0.05).

To evaluate the functional role of TRIP13 in myeloma pathogenesis, we overexpressed TRIP13 in the MM cell lines ARP1, OCI-MY5, and H929 using lentivirus-mediated human TRIP13-cDNA (Figure 2A). The cell number in all three TRIP13-overexpressing (OE) cell lines significantly increased after 3-day cultures, indicating that high levels of TRIP13 promote MM cell growth (Figure 2B, p < 0.05).

To explore the effects of TRIP13 on drug resistance in MM, TRIP13-OE MM cell line ARP1 and OCI-My5 or empty vector-transfected cells were treated with different concentrations of anticancer reagents: proteasome inhibitor Bortezomib or topoisomerase inhibitor etoposide. Cell viability assay was performed 24 hours later using the PrestoBlue Cell Viability reagent. We observed that the number of viable cells in TRIP13-OE ARP1 and OCI-My5 cells was significantly higher after drug treatment compared with empty vector control cells (Figure 2C and 2D, p < 0.05). To examine whether drug resistance was related to decreased apoptosis, we performed FITC-conjugated annexin V/Hoechst 33258 staining inTRIP13-OE MM cells or empty vector cells treated with Bortezomib. Our results indicated that overexpression of TRIP13 led to decreased apoptosis and conferred protection against drug-induced cytotoxicity in myeloma cells when treated with increasing doses of Bortezomib (5–20 nM) (Figure 2E, p < 0.05). Consistently, Bortezomib-induced G2/M cell cycle arrest was inhibited in TRIP13-OE MM cells compared to those control cells (Figure 2F, p < 0.05).

### TRIP13 plays an oncogenic role using *in vitro* colony assay and *in vivo* NOD-Rag/null gamma mice

**Figure 3:**
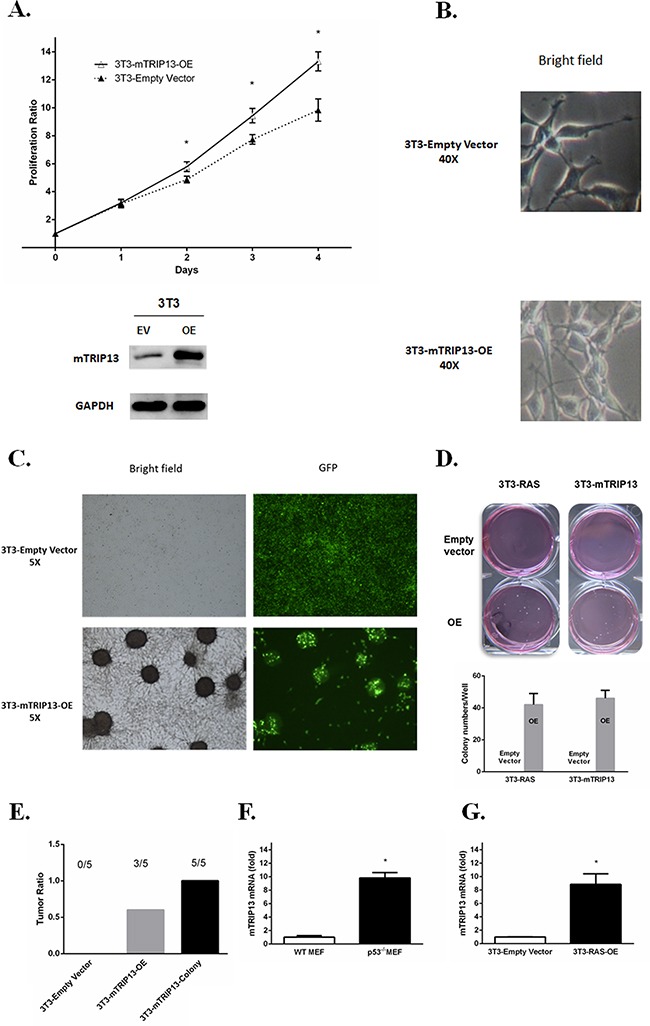
TRIP13 promotes tumorigenesis in NOD-Rag/null gamma mice **A**. Murine Tripl3 (mTRIP13) overexpression was identified in NIH/3T3 cells transfected with mTRIP13 overexpressing plasmids (OE) compared to those transfected with empty vector (EV) (below panel). Cells were counted by trypan blue exclusion for consecutive 4 days (upper panel). Overexpression of mTRIP13 resulted in increased cell growth. Results from 3 independent experiments are shown (*p < 0.05). **B**. Morphology of NIH/3T3-Empty Vector or NIH/3T3-mTRIP13-OE cells was shown in liquid media under microscope with bright field (magnification×40). **C**. Overall growth of NIH/3T3-Empty Vector or NIH/3T3-mTRIP13-OE cells was shown in liquid media under microscope with bright field and green fluorescence (GFP) (magnification×5). **D**. Representative images of overall colony formation of NIH/3T3-mTRIP13-EV or NIH/3T3-mTRIP13-OE cells along with NIH/3T3 cells transfected with KRAS overexpressing plasmids (3T3-RAS-OE) or empty vector (3T3-RAS-EV) in each well were shown (upper panel). Colony-forming numbers were quantified with software ImageJ and results were from 3 independent experiments (below panel). **E**. NIH/3T3-Empty Vector or NIH/3T3-mTRIP13-OE, as well as the isolated colony-forming cells (3T3-mTRIP13-Colony) were subcutaneously injected (2.5×10^5^ cells/flank) into NOD/SCID mice and assessed for tumor formation on day 26 post injection. **F**. The mTRIP13mRNA level was almost 10-fold higher in p53 knockdown mouse embryo fibroblast (p53^−/−^MEF) compared to wild type MEF (WT MEF). Results from 3 independent experiments are shown(*p <0.05). **G**. The mTRIP13mRNA level was 8-fold higher in KRAS mutated and overexpressed NIH/3T3 (3T3-RAS-OE) compared to control cells transfected with empty vector. Results from 3 independent experiments are shown (*p <0.05).

TRIP13 was elevated from MGUS to MM patient samples, suggesting that it may get involved in tumorigenesis. Human and murine TRIP13 (mTRIP13) proteins share a sequence identity of 93%, as assessed by a blast search. To clarify the role of TRIP13 in malignant cellular transformation, we overexpressed mTRIP13 in mouse nontransformed fibroblasts NIH/3T3. As a result, overexpression of mTRIP13 increased cell growth (Figure 3A) with a transformed phenotype of a spindle morphology, refractivity and multilayered growth (Figure 3B, below). While the control cells transfected with empty vector displayed growth in monolayers, resembling parental cells in morphology (Figure 3B, upper). In culture flask, we observed anchorage-dependent colonies with green fluorescence (mTRIP13 conjugated with GFP) in mTRIP13-OE but not in empty vector fibroblasts (Figure 3C). We then performed and compared anchorage-independent colony formation in soft agar between mTRIP13-OE and EV NIH/3T3 cells, using a KRAS mutated (V12, activating mutant) and overexpressed NIH/3T3 cells as a positive control. Similar to RAS-mediated colony transformation, mTRIP13-OE also exhibited the capacity of clonogenesis. After a 2-week culture, we observed 40–50 colonies in each well of 6-well plate with mTRIP13-OE, whereas no pronounced colony was found plated with control cells (Figure 3D). We then selected one of the mTRIP13-OE clones from soft agar and independently expanded it in liquid media for further analysis. To assess the role of mTRIP13 in tumorigenesis *in vivo*, we subcutaneously injected 2.5 × 10^5^ mTRIP13-OE or empty vector-transfected NIH/3T3 cells, or cells isolated from colonies into each flank of NOD/SCID mice, and evaluated tumor growth respectively. As a result, tumor mass was palpable on day 17 post injection in colony-isolated cells, on day 19 in mTRIP13-OE cells, but was not obviously palpable in empty vector-transfected control cells on day 26 post injection. Totally, all mice with colony-isolated cells developed tumors. Of the mice injected with mTRIP13-OE cells, 60% (three of five) developed tumors. By contrast, none of the mice injected with empty vector-transfected cells had tumors (Figure 3E). It is also well known that p53 deletion and mutations are involved in MM pathogenesis [11], we thus compared the mTRIP13 mRNA between p53^−/−^ mouse embryo fibroblast cells (MEF) and wild type MEFs. The mTRIP13 was almost 10-fold higher in p53^−/−^ MEF (Figure 3F) and 8-fold higher in KRAS mutated and overexpressed NIH/3T3 cells (Figure 3G) compared to their counterparts, respectively.

### Knockdown of TRIP13 inhibits myeloma cell growth *in vitro* and *in vivo*

**Figure 4:**
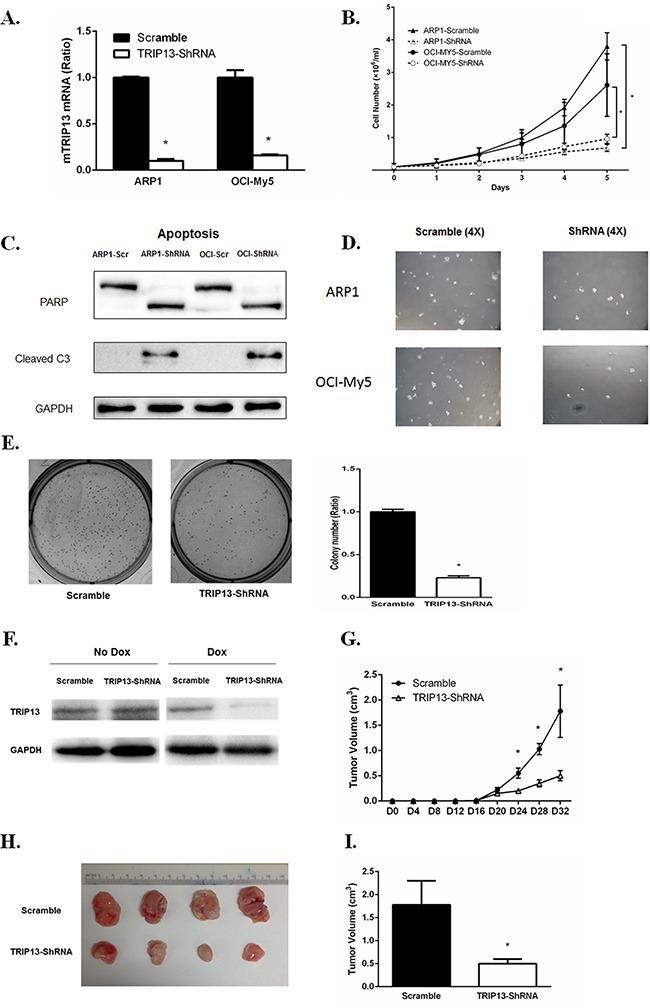
TRIP13 knockdown inhibits myeloma cell growth, induces apoptosis and lowers tumor burden *in vivo* **A**. TRIP13 knockdown was identified in myeloma cell lines ARP1 and OCI-My5 (TRIP13-ShRNA) compared to control cells that were non-target scramble transfected (Scramble). **B**. TRIP13-ShRNAMM cell lines ARP1, OCI-My5 as well as their scrambles were counted by trypan blue exclusion for consecutive 5 days for cell growth. Results from 3 independent experiments are shown (*p < 0.05). **C**. PARP and cleaved caspase 3 (C3) were determined using western blot in TRIP13-ShRNAmyeloma cell lines ARP1 and OCI-My5 and their scrambled controls (Scr) 48 h post Doxycycline (Dox) treatment. **D**. Representative images of anchorage-independent colony formations of TRIP13-ShRNA myeloma cell lines ARP1 and OCI-My5 and their scrambled controls (Scramble) (magnification×4). **E**. Representative images of overall colony formation for TRIP13-ShRNA myeloma cell lines ARP1 and the scrambled control (Scramble) in the each well (left panel). Overall colony numbers of each well were quantified and the percentage of TRIP13-ShRNA/Scramble was calculated. Results from 3 independent experiments are shown (*p<0.05) (right panel). **F**. Dox treatment induced effective TRIP13 knockdown in tumor masses of mice injected with TRIP13-ShRNA myeloma cell line OCI-My5 compared to those with scrambled control (Scramble). **G**. 1.5×10^6^ TRIP13-ShRNA OCI-My5 cells or the scrambled control (Scramble) were injected subcutaneously into the abdomen of NOD-Rag/null gamma mice. TRIP13 knockdown was induced by the addition of Dox to the drinking water 16 days after injection. Tumor growth was assessed for each group every four days (*p < 0.05). **H**. Mice were properly sacrificed and differences in tumor size were shown between the two groups of mice on Day 32 post injection. **I**. Tumor volume was quantified and compared between the two groups of mice (*p<0.05).

We designed three independent TRIP13-ShRNA sequences, which target discrete coding regions of TRIP13, resulting in an approximately 80% knockdown of TRIP13 mRNA upon doxycycline (Dox) treatment for the most effective one (Figure 4A). ARP1 and OCI-MY5 MM cell lines were infected TRIP13-ShRNA by lentiviral delivery. Cell growth was monitored daily for 5 consecutive days after TRIP13 knockdown (48 h post Dox treatment) and nontarget scramble (SCR)-transfected cells were used as controls. Data showed that silencing of TRIP13 significantly inhibited MM cell growth in both ARP1 and OCI-MY5 cell lines compared to the scrambled controls (Figure 4B, p < 0.05). To determine whether apoptosis contributed to TRIP13-ShRNA mediated cell growth inhibition, we examined PARP and cleaved caspase 3 by western blotting. TRIP13 knockdown led to pronounced apoptosis in ARP1 and OCI-MY5 after 48 hours of Dox treatment (Figure 4C). We also performed a colony formation assay in TRIP13-ShRNA MM cells. Knockdown of TRIP13 decreased anchorage-independent colony number in both ARP1 and OCI-MY5 cell lines (Figure 4D). The Figure 4E presented the whole well colonies and showed that the number is significantly decreased in TRIP13-ShRNA ARP1 cells compared to scramble ones (p < 0.05).

We next determined the effects of TRIP13-ShRNA on MM cell growth *in vivo*. OCI-MY5 MM cells transduced with TRIP13-ShRNA or scrambled vectors were injected subcutaneously into the abdomen of NOD Rag/null gamma mice (five mice in each group). Dox was added into the drinking water 16 days post tumor engraftment to induce TRIP13 knockdown, which was validated from tumor masses (Figure 4F). Tumor growth of each group was evaluated every four days (Figure 4G). Mice were properly sacrificed and tumors were dissected on Day 32 (Figure 4H). We observed that, tumor size was significantly smaller in the TRIP13-ShRNA mice compared to those controls (Figure 4I, p < 0.05).

### Overexpression of TRIP13 abrogates the spindle checkpoint

**Figure 5:**
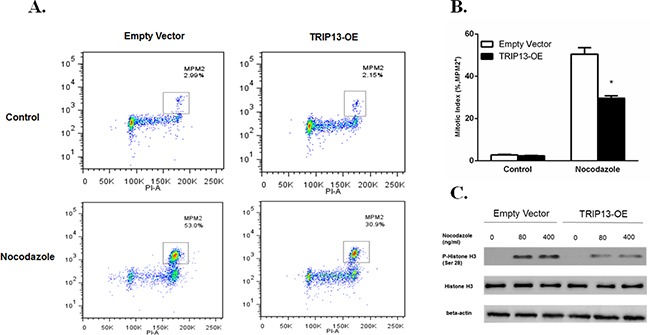
Overexpression of TRIP13 abrogated the spindle checkpoint **A**. TRIP13-OE MM cell line ARP1 or empty vector-transfected cells were treated with 100 ng/ml Nocodazole for 18 hours, stained with α-MPM2 and PI and then analyzed by FCM. The populations of mitotic cells (with 4N DNA content and MPM2 staining) were boxed with the mitotic indices indicated. **B**. Quantification of mitotic indices of cells described in A. Results from 3 independent experiments are shown (*p < 0.05). **C**. Western blot of phospho-Histone H3 and overall Histone H3 in TRIP13-OE MM cell line ARP1 or empty vector-transfected cells treated with indicated concentrations of Nocodazole for 18 h.

TRIP13 plays an important role in chromosomal structure development during mitosis [9]. Mitosis-specific phosphor-proteins include topoisomerase II alpha, microtubule associated protein, NimA and two Cdc2- inhibitory kinases. These proteins sharing epitopes that are phosphorylated during the induction of mitosis could be recognized by monoclonal antibody mitotic protein monocolonal 2 (MPM2), which is regarded as a mitotic marker [12]. To evaluate if TRIP13 plays a role in MM mitosis, TRIP13-OE and empty vector ARP1 cells were treated with the spindle toxin 100 ng/ml nocodazole for 18h. MM cells were then stained with propidium iodide (PI) and MPM2, and analyzed by FCM. MM cells having 4N DNA content and being MPM2-positive concurrently indicate mitosis. Clearly, Tripl3-OE ARP1 cells showed much less mitotic cells compared to those control cells treated with nocodazole (Figure 5A and 5B, p < 0.05), suggesting that high-level TRIP13 could deactivate spindle checkpoint and halt mitotic progression in the presence of spindle poisons. Furthermore, phosphorylation of histone H3 at Ser10 is tightly correlated with chromosome condensation during mitosis and is considered as another mitotic marker [13]. Consistently, TRIP13-OE also decreased the levels of phospho-histone H3 in ARP1 cells treated with the indicated concentrations of nocodazole (Figure 5C).

### Overexpression of TRIP13 induces proteasome degradation of MAD2 through Akt pathway

**Figure 6:**
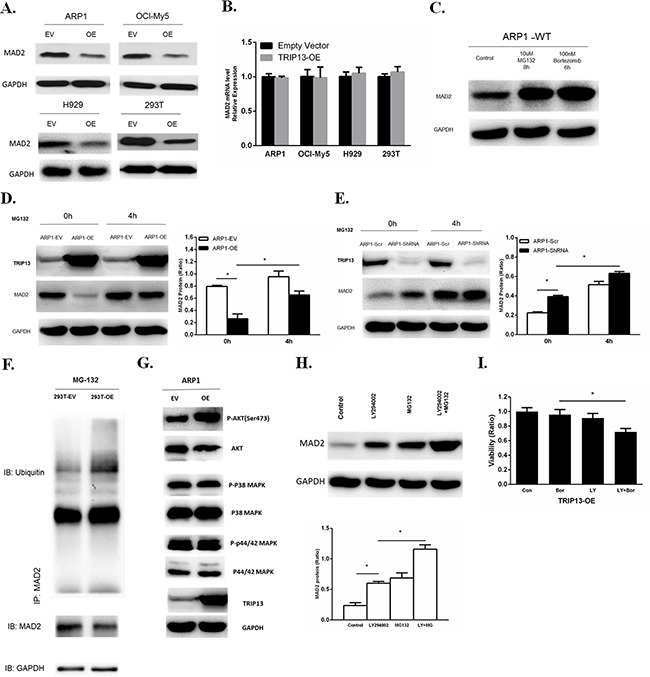
Overexpression of TRIP13 induces proteasome-mediated MAD2 degradation through Akt pathway **A**. Western blot of MAD2 in human TRIP13 overexpressing myeloma ARP1, OCI-My5, H929 cells and HEK293T cells (OE) as well as their controls transfected with empty vectors (EV). **B**. Realtime PCR analysis of MAD2 mRNA in human TRIP13 overexpressing myeloma ARP1, OCI-My5, H929 cells and HEK293T cells (TRIP13-OE) as well as their controls transfected with empty vectors. Results from 3 independent experiments are shown (OE *vs*. Empty Vector, p > 0.05). **C**. Western blot of MAD2 in wild type ARP1 (ARP1-WT) cells treated with proteasome inhibitor MG132 and Bortezomib for indicated period of time. **D**. Western blot of MAD2 in TRIP13 overexpressing ARP1 cells (ARP1-OE) and control cells transfected with empty vectors (ARP1-EV) treated with proteasome inhibitor MG132 for indicated period of time. Bands of MAD2 have been quantified. Results from 3 independent experiments are shown (*p < 0.05). **E**. Western blot of MAD2 in TRIP13 knockdown ARP1 cells (ARP1-ShRNA) and the scrambled control (ARP1-Scr) treated with proteasome inhibitor MG132 for indicated period of time. Bands of MAD2 have been quantified and data. Results from 3 independent experiments are shown (*p < 0.05). **F**. Co-immunoprecipitation (Co-IP) demonstrated ubiquitination of MAD2 was increased in TRIP13 overexpressing HEK293T cells (293T-OE) compared to control cells transfected with empty vectors (293T-EV). **G**. Western blot of signaling pathways including Akt, P38 MAPK, P44/42 MAPK in TRIP13 overexpressing ARP1 cells (OE) and control cells transfected with empty vectors (EV). **H**. Western blot of MAD2 in TRIP13 overexpressing ARP1 cells treated with10 μMPI3K/Akt inhibitor LY294002 for 12 h or 10 μMMG132 for 4 h independently, or with the combination of LY294002 and MG132 (LY+MG). Bands of MAD2 have been quantified. Results from 3 independent experiments are shown (*p < 0.05). **I**. TRIP13 overexpressing ARP1 cells (TRIP13-OE) were pretreated with or without 10 μMLY294002 (LY) for 2 hours and then cells were treated with 5 nM bortezomib (Bor) for 24 h. Cell viability assay was performed. Results from 3 independent experiments are shown (*p < 0.05).

Spindle checkpoint proteins are critical to the mitotic arrest. MAD2 is a key component of the spindle checkpoint; it binds to and inhibits the APC/C activator CDC20, and prevents the precocious onset of anaphase by inducing mitotic arrest in the presence of spindle poisons [14]. We performed western blots and observed that overexpression of TRIP13 decreased MAD2 protein not only in human MM cell lines ARP1, OCI-MY5 and H929, but also in HEK293T cells (Figure 6A). However, the decreased levels of MAD2 protein were not consistent with MAD2 mRNA expression, which was not significantly changed in TRIP13-OE cells compared to control cells (Figure 6B). Moreover, MAD2 protein stability has been assessed using cycloheximide to inhibit protein synthesis. Consistent decreases in MAD2 were observed in TRIP13-OE ARP1 cells (Supplementary Figure 1). Therefore, we hypothesized that TRIP13 regulates MAD2 expression in a post-translational modification manner. To test this hypothesis, MAD2 protein was analyzed in the myeloma ARP1 cells treated with a proteasome inhibitor either MG132 or Bortezomib. Results demonstrated MAD2 was significantly elevated upon proteasome inhibition, suggesting that MAD2 protein is indeed degraded in proteasomes (Figure 6C). Furthermore, the MAD2 protein was partially rescued after addition of MG132 for 4 hours in TRIP13-OE ARP1 cells (Figure 6D). While the MAD2 protein was elevated in the ARP1 cells knocked down TRIP13, which was similar to the MG-132 treatment (Figure 6E). The similar results were observed in 293T cells (Supplementary Figure 3). These data support that high levels of TRIP13 increase the degradation of MAD2 protein.

We further determined whether TRIP13 increases MAD2ubiquitination. The lysates from HEK293T cells with or without TRIP13-OE were co-immunoprecipitated with the MAD2 antibodies in the presence of MG132. The ubiquitinatedMAD2 was detected by anti-ubiquitin antibody. As shown in the Figure 6F, TRIP13-OE significantly increased the ubiquitination of MAD2 protein (Figure 6F). Western blots were also performed to examine the expression of p-Akt, p-P38 MAPK and p-P44/42 MAPK in TRIP13-OE ARP1 cells, we found that the phosphorylated Akt was obviously increased in TRIP13-OE cells (Figure 6G). However, phosphorylation of P38 and p-P44/42 MAPK were not significantly changed after TRIP13 overexpression. Further, we demonstrated a PI3K/Akt inhibitor LY294002 significantly elevated MAD2 levels at 12 h in TRIP13-OE ARP1 cells. In addition, the combination of MG132 with LY294002 further increased the MAD2 protein abundance, suggesting an additive role of proteasome inhibition (Figure 6H). Furthermore, PI3K/Akt inhibition by LY294002 pretreatment partially restored cell sensitivity to Bortezomib in TRIP13-OE myeloma cells, although LY294002 alone had slightly inhibitive effects (Figure 6I). Therefore, these results indicate that the Akt pathway is possibly required for MAD2 degradation and drug resistance in TRIP13-OE cells.

## DISCUSSION

As early as 2006, TRIP13 has been identified as one of the 25 chromosome instability (CIN) genes by analyzing 18 solid tumor data sets [15]. Consistently, we recently found that CIN-related genes, including TRIP13, are associated with drug resistance and poor outcomes in MM [5].

In this study, we further demonstrate in detail that TRIP13 increases with the progression of MM, while high level TRIP13 relates to relapsed MM patients and shortened OS. These clinical data support that TRIP13 may play a critical role in MM pathogenesis. Since human TRIP13 has two isoforms: isoform 1 with 432 amino acids (AA) and isoform 2 with 289 AA which has a shorter C-terminus, we specified isoform 1 as the TRIP13 expression signal derived from the GEP data by analyzing sequences of the 11 probe sets from Affymetrix U133Plus2 platform. Therefore, all studies applied in this project are designated for the long isoform of TRIP13.

TRIP13, a mouse orthologue of pachytene checkpoint 2 (Pch2), is required during meiotic double-strand break repair and is critical in the regulation of the meiotic chromosome structure and recombination in the yeast *Caenorhabditiselegans* [16, 17]. Human TRIP13 has been reported to be a novel kinetochore protein, which directly interacts with mitotic checkpoint silencing protein p31 and plays an important role in mitosis to achieve faithful chromosome segregation [18]. Also, we have sequenced the genomes of the TRIP13-overexpressing MM cell line ARP1 (ARP1-OE) and empty vector-transfected control cells. By DNA copy number variations (CNVs) analysis we identified 102 putative CNV events occurring in the ARP1-OE cells (Supplementary Figure 3), providing direct evidence that TRIP13 is involved in chromosome stability. However, mis-segregation of chromosomes and aneuploidy are often associated with the development of cancers. High TRIP13 expression has been identified in multiple cancers, including prostate cancer [19], primary cutaneous T-cell lymphoma [20], non-small cell lung cancer [21], and breast cancer [22]. Furthermore, the high expression of TRIP13 has been associated with aggressive subtype, treatment resistance, and enhanced repair of DNA damage in head and neck cancer [10]. The same study that used a chick chorioallantoic membrane (CAM) assay suggested that the overexpression of human TRIP13 in mouse fibroblasts NIH/3T3 cells resulted in focus formation, cell proliferation, and tumor growth *in vivo* [10]. Our GEP data also show TRIP13 is significantly increased in MM patients compared to NPC and MGUS samples, providing clinical evidence that TRIP13 could be oncogenic. Consistently, we found that murine TRIP13, which shares 93% protein identity with human TRIP13, could also lead to malignant cell transformation both *in vitro* and *in vivo* when overexpressed in non-malignant NIH/3T3 cells. These results confirm that TRIP13 has an oncogenic potential. P53 has been demonstrated to mediate cancer stem–like cell function by inhibiting pluripotency and cellular dedifferentiation. P53 deletion has been identified in over 10% of newly diagnosed MM patients and is regarded as a poor prognostic factor [11]. In addition, the oncogenic RAS has been shown to play an important role for MM cell survival [23] and indicates refractory disease [24]. Interestingly, in our study, the observation that TRIP13 was higher in p53^−/−^NIH/3T3 and also in KRAS mutated MEFs indicates TRIP13 could be a downstream factor regulated by p53 and/or KRAS. However, the detailed network among p53, RAS and TRIP13 in their oncogenic network needs further study.

Drug-resistant subclones in MM, either present at diagnosis or developed after chemotherapy treatment, are responsible for treatment failure resulting in MM relapse and patient death. The observations based on GEP data, that increased TRIP13 expression in patients with poor survival and again in relapsed MM samples, suggest that TRIP13 may play a major role in MM progression. We found indeed high TRIP13 increases MM cell growth and drug resistance *in vitro*, while knockdown of TRIP13 inhibits MM cell survival. Previously, we have shown that *NEK2* upregulates the expression of drug efflux pumps such as MDR1 (ABCB1) and MRP1 (ABCC1) [5]. However, we did not find such molecular alteration in TRIP13-OE cells (data not shown), indicating that other mechanisms involved.

Dysfunction of checkpoint proteins leads to impairments of DNA damage repair and subsequent CIN. Spindle checkpoint proteins, also called mitotic checkpoint complex (MCC), consist of the *MAD2*, *BubR1*, and *Bub3*, which secure accuracy of chromosome segregation [25]. Defects in MCC surveillance system contribute to chromosome mis-segregation, aneuploidy and a failure to arrest in mitosis in the presence of microtubule poisons, ultimately leading to the development of human cancers and drug resistance in cancer [2]. Recently, it has been demonstrated that TRIP13, in conjunction with the MAD2-binding protein p31^comet^, inactivates MCC, presenting as a mitotic checkpoint-silencing protein [4, 26]. In worms, TRIP13, an AAA+ ATPase, was found to catalyze MAD2 from a signaling-active closed conformer to an inactive open conformer to disassemble and inactivate MCC [16]. In this study, we show that overexpression of TRIP13 decreases MAD2 protein in human MM cell lines and HEK293T cells, as well as in murine fibroblast NIH/3T3 cells (data not shown). MAD2 (mitotic arrest deficiency protein 2), a key component for regulating MCC surveillance system, plays an inhibitory role in anaphase-promoting complex/cyclosome (APC/C) by counteracting the effect of CDC20 [14]. When the MCC surveillance system is turned on, MAD2 forms a complex with CDC20 and APC/C, inactivates securin and separase, and consequently arrests cells at prometaphase. Conversely, the reduced expression of MAD2 protein results in attenuated MCC surveillance and subsequent CIN and drug resistance [27]. The down-regulation or deletion of MAD2 also has been reported in a variety of human cancers. Moreover, downregulation of MAD2 is shown to increase proliferation and enhance the drug resistance in gastric cancer cells by increasing expression of phosphorylated survivin [28]. However, the mechanisms underlying the downregulation of MAD2 have not been fully elucidated. Our study of MM model, for the first time, brings out that decreased MAD2 is in part caused by high TRIP13 enhanced MAD2 ubiquitination and proteasome degradation, although it is not clear how TRIP13 promotes MAD2ubiquitination. Considering the role of TRIP13 in the conformational change of MAD2 to inactivate MCC in worms [16], we propose that TRIP13 possibly has a dual-effect on the inhibition of MAD2 to dissemble MCC and attenuate the surveillance system. PI3K/Akt signaling pathway plays a critical role in the regulation of survival, proliferation, migration, angiogenesis, and drug resistance of MM cells [29]. Also, PI3K/Akt activation occurs during the ubiquitination and degradation of other proteins to regulate tumorigenesis and chemoresistance [30, 31]. We also demonstrate that the Akt pathway mediates MAD2 degradation induced by TRIP13 and PI3k/Akt inhibition could in part revert drug resistance to Bortezomib. It suggests that the Akt inhibitor, which has been used in clinic [32], may be functional to target TRIP13 related tumor features.

Taken together, TRIP13 ubiquitinates and degrades the checkpoint surveillance protein MAD2 via activating Akt signaling pathway, leading to weakened checkpoint surveillance and consequent tumorigenic aneuploidy and drug resistance in MM. Our data suggest that targeting TRIP13 may provide a novel treatment to overcome drug resistance in MM.

## MATERIALS AND METHODS

### Samples from patients with multiple myeloma and accession numbers

To analyze the expression data of TRIP13 and associated clinical information, all the microarrays were performed on primary myeloma samples. The Gene Expression Omnibus database accession numbers described in this article were retrieved from GSE19554, GSE24080, GSE2658, GSE31161, GSE5900 and GSE6205. Kaplan-Meier analyses of overall survival (OS) about patients in the TT2 and TT3 cohorts were performed using methods implemented in the R package survival. All other statistical analyses, including the evaluation about the statistical significance of differences in TRIP13 expression between patient groups were performed using student's t-test. The probe sequences of Tripl3 signature were obtained from Affymetrix official website (www.affymetrix.com) and have been identified specific to the non-coding sequence of TRIP13 isoform 1 by sequence blast.

### Cell growth and viability assay

Myeloma cells were grown in RPMI1640 (Gibco) at 37°C, 5% carbon-dioxide and saturated humidity. The media were supplemented with 10% fetal calf serum, 100 U/ml penicillin, 100 mg/ml streptomycin and 2 mM L-glutamine (Gibco). For cell growth, the live cells were counted by trypan blue dye exclusion assay for 3-5 days. Cell viability was detected using PrestoBlue Cell viability reagent (Life Technologies) according to the manufacturer's instructions and presented as the percentage relative to control cells.

### Constructs of letivirus plasmids for TRIP13 overexpression and conditional knockdown, quantitative real-time PCR

pCDH and pCAG/AIG constructs were used to generate human and murine TRIP13 overexpression plasmids respectively. Packaging plasmid VSVG and psPAX2 are required to produce recombinant lentivirus. The nucleotide sequences of the primers were as follows: Human TRIP13, sense: *5′-AAAATCTAGAATGGACGAGGCCGTGG-3′*, antisense: *5′-AAAAGGATCCTCAGATGTAAGCTGCAAGC-3′* (accession number NM_004237.3). Murine TRIP13, sense: *5′-AAATCTAGAATGGATTACAAGGACGACGATGACAAGGACGAGGCGGTGGGCGACC-3′*, antisense: *5′-AAAGGATCCTCAAACATAAGCTGAAAGTTTCTTTTTCTCCTCAAACTG-3′* (accession number NM_027182.2). In addition, pTripZ-TRIP13-ShRNA was constructed to generate conditional knockdown of human TRIP13. Three different synthetic oligonucleotide sequences specific for TRIP13 silencing (ShRNA) were designed, with one identified as most effective shown in the following: *5′-GTTGACAGTGAGCGACACCAAGGCCAGGCTTTGTTATAGTGAAGCCACAGATGTATAACAAAGC CTGGCCTTGGTGGTGCCTACTGCCT-3′*. A nonsense scrambled oligonucleotide was used as a control. Recombinant lentivirus was produced by transient transfection of HEK293T cells using packaging plasmid VSVG and psPAX2. Transfection of myeloma cell lines by lentivirus was performed using polybrene. Transduced cell lines were selected in puromycin. After 3 days of selection, puromycin was removed and efficient knock-down of TRIP13 were induced 48 hours post Doxycycline (Dox, 10ng/ml) treatment. All constructed plasmids carry fluorescence proteins. Efficiency of viral transfection was > 95% determined by percentage of transfected cells with fluorescence using flow cytometry. As for *in vivo* TRIP13 knockdown, Dox (2mg/ml) was added into the drinking water 16 days post tumor engraftment. Experimental procedures for quantitative real-time PCR with target genes were described before [33].

### Co-immunoprecipitation assay and western blot analysis

10^7^ cells were washed once in PBS, and lysed in 1ml lysis buffer (pierce 87788) containing protease and phosphatase inhibitor (pierce 88669), and centrifuged for 10min at 16000 rpm at 4°C. The supernatant was transferred to a fresh tube. MAD2 proteins were immunoprecipitated with proteinA/G beads covalently linked to an anti-MAD2 antibody. Experimental procedures for co-immunoprecipitation are as described [34]. After SDS–PAGE, the samples were transferred to a nitrocellulose membrane. Western blotting analysis was performed with anti-ubiquitin antibody and other indicated antibodies as described before for detailed procedures [33].

### Soft agar assay of colony formation

1×10^4^ NIH/3T3 cells transduced with control vector or murine TRIP13 (mTRIP13) were mixed with RPMI1640 media containing 10% FBS and 0.33% agar and layered on top of the base layer of 0.5% agar in each well of 6-well plates. Colony numbers were counted after approximately 2 weeks. Colony cells were isolated with a 1-ml pipet and transferred to 12-well plates for further analysis. For the myeloma line ARP1 and OCI-MY5 cells with TRIP13 knockdown, the same colony-forming method was applied. All plates were pictured under microscope and overall numbers of colony in the pictures were scanned and counted by the Image J software.

### Murine model of tumorigenesis and human myeloma in NOD-Rag/Null gamma mice

All animal work was performed under approved protocol in accordance with the guidelines of the Institutional Animal Care and local veterinary office and ethics committee of Tongji University. Each group consisted of five mice. For tumorigenesis assay, 2.5×10^5^ NIH/3T3 cells transduced with control vector or mTRIP13, or colony cells isolated from mTRIP13 overexpressing NIH/3T3 cells were injected subcutaneously into each side of mice dorsa. Tumor incidence and the number of tumor nodules from each group were counted and compared among them. For murine model of human myeloma, myeloma line OCI-MY5 cells were transduced with TRIP13-ShRNA or scrambled (SCR) control vectors. 1.5×10^6^ cells (in 100 ml PBS) were injected subcutaneously into the abdomen of NOD-Rag/null gamma mice. TRIP13 knockdown was induced by the addition of doxycycline (2 mg/ml) to the drinking water 16 days after injection when tumor masses were palpable. Tumor length and width were gauged, and tumor volume was calculated as (length × width^2^) × 0.5. For each time point, results are presented as the mean tumor volume ± SD for the indicated mice.

### Flow cytometry (FACS)

For apoptosis analysis, cells were stained with Annexin V/PI (BD Pharmingen) according to the product instructions. Cells were then analyzed for apoptosis by FACS using the Cell Quest software. For analysis of cell cycle, cells were washed and fixed in 70% ethanol overnight at −20°C, treated with RNaseA (Sigma, USA), stained with propidium iodide (Sigma), and assayed for DNA content by FACS. The acquired histograms were analyzed by ModFit LT software to determine the cell cycle phase distribution. For the evaluation of mitosis, cells were arrested at metaphase with 100 ng/ml nocodazole (Sigma) for 18 h prior to harvesting. After washed once with PBS, cells were fixed with 70% cold ethanol for overnight fixation at −20°C. Permeabilized with a pre-incubation of Triton X-100, cells were then incubated with α-MPM2 (Millipore) followed by fluorescence-conjugated secondary antibodies (Invitrogen). After washed with PBS once, cells were stained with propidium iodide and then analyzed by FACS with FacsScan (Becton Dickinson). The results were studied using the FlowJo software.

### Statistical analysis

All data were shown as means±SD. The Student's t test was applied to compare two experimental groups. The relevance of TRIP13 expression to disease progression, event-free survival (EFS) and overall survival (OS) was measured using the Kaplan-Meier method. The log-rank test was used for group comparison. Significance was set at p < 0.05.

## SUPPLEMENTARY FIGURES


