# Supplementary material for: TRIP13 impairs mitotic checkpoint surveillance and is associated with poor prognosis in multiple myeloma
Source: Oncotarget. 2017 Feb 1;8(16):26718–31. doi: 10.18632/oncotarget.14957 (PMC5432292; doi:10.18632/oncotarget.14957)
Supplement: Supplementary file 1 [file oncotarget-08-26718-s001.pdf]

TRIP13 impairs mitotic checkpoint surveillance and is associated with poor prognosis in multiple myeloma

SUPPLEMENTARY FIGURES

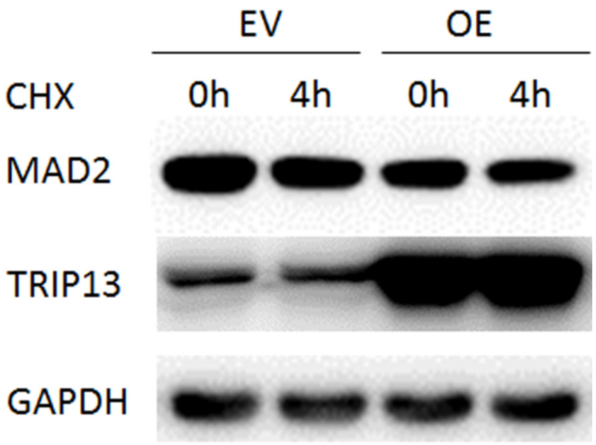

Supplementary Figure 1: TRIP 13 overexpressing (OE) ARP1cells and their counterparts transfected with empty vectors (EV) were treated with 20 mg/ml cycloheximide (CHX) for 4 h before harvest, and endogenous MAD2 protein levels were detected by western blotting.

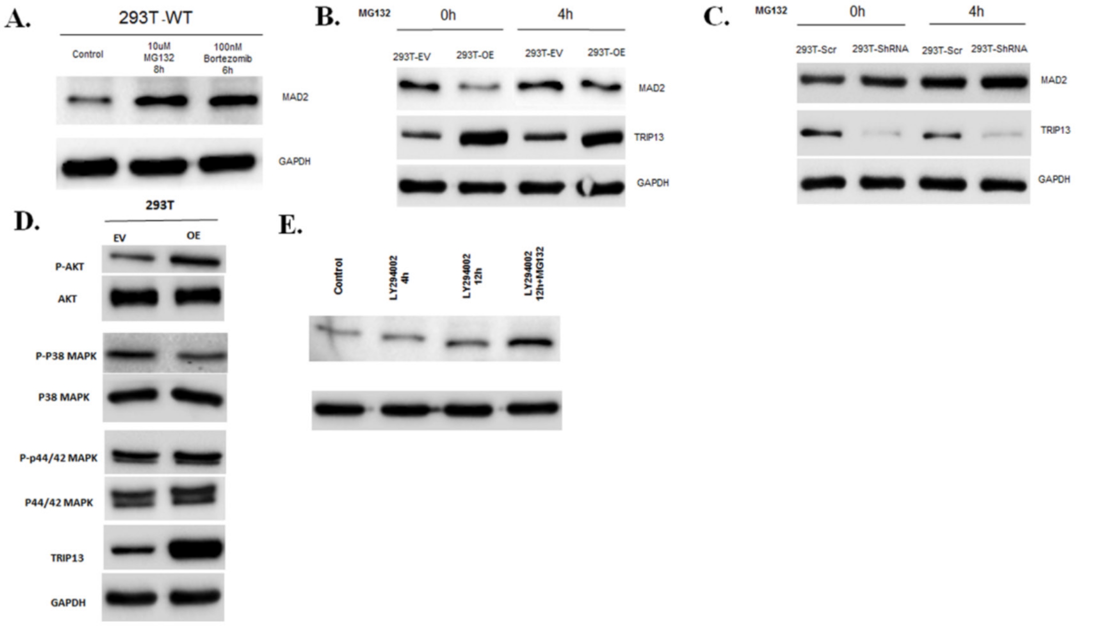

Supplementary Figure 2: A. Western blot of MAD2 in wild type HEK293T (293T-WT) cells treated with proteasome inhibitor MG132 and Bortezomib. **B.** Western blot of MAD2 in TRIP13 overexpressing HEK293T cells (293T-OE) and control cells transfected with empty vectors (293T-EV) treated with MG132. **C.** Western blot of MAD2 in TRIP13 knockdown HEK293T cells (293T-ShRNA) and the scrambled control (293T-Scr) treated with MG132. **D.** Western blot of signaling pathways including Akt, P38 MAPK, P44/42 MAPK in 293T-EV and 293T-OE. **E.** Western blot of MAD2 in 293T-OE treated with PI3K/Akt inhibitor LY294002 independently or in combination with MG132 for the last 4 hours.

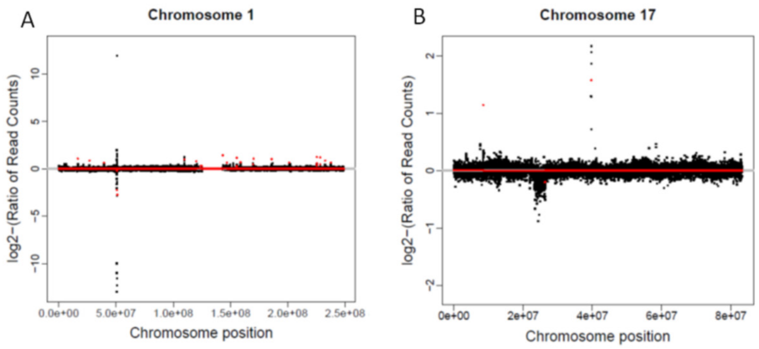

Supplementary Figure 3: DNA copy number variation analysis about the MM cell line ARP1 overexpressing TRIP13 (ARP1-OE) and ARP1 cells transfected with empty vector (ARP1-EV). Y-axis represents log2-transformed ratio of read counts (black points), and estimated log2-(fold change) of DNA copy numbers, for ARP1-OE relative to ARP1-EV. Red line indicates the average ratio of copy numbers across the chromosome. Chromosome 1 **A.** and 17 **B.** are shown here for demonstration. Note that ARP1-OE and ARP1-EV were cultured for only one generation after transfection and before submission for high-throughput DNA sequencing.
